# Long-term survival and predictors for mortality among dialysis patients in an endemic area for chronic liver disease: a national cohort study in Taiwan

**DOI:** 10.1186/1471-2369-13-43

**Published:** 2012-06-18

**Authors:** Chih-Chiang Chien, Jhi-Joung Wang, Yih-Min Sun, Ding-Ping Sun, Ming-Jen Sheu, Shih-Feng Weng, Chin-Chen Chu, Hung-An Chen, Chung-Ching Chio, Jyh-Chang Hwang, Yi-Hua Lu, Hsien-Yi Wang, Wei-Chih Kan

**Affiliations:** 1 Department of Nephrology, Chi-Mei Medical Center, No.901, Zhonghua Rd, Tainan City, Yongkang Dist 710, Taiwan; 2 Department of Food Nutrition, Chung Hwa University of Medical Technology, Tainan, Taiwan; 3 Department of Medical Research, Chi-Mei Medical Center, Tainan, Taiwan; 4 Department of Occupational Safety and Health, Chung Hwa University of Medical Technology, Tainan, Taiwan; 5 Division of Transplantation, Chi-Mei Medical Center, Tainan, Taiwan; 6 Chia Nan University of Pharmacy and Science, Tainan, Taiwan; 7 Department of Gastroenterology, Chi-Mei Medical Center, Tainan, Taiwan; 8 Department of Rheumatology, Chi-Mei Medical Center, Tainan, Taiwan; 9 Department of Neurological Surgery, Chi-Mei Medical Center, Tainan, Taiwan; 10 Department of Medical Laboratory Science and Biotechnology, Chung Hwa University of Medical Technology, Tainan, Taiwan; 11 Institute of Biomedical Engineering, Southern Taiwan University, Tainan, Taiwan

**Keywords:** Hemodialysis, Peritoneal dialysis, Mortality, Liver cirrhosis

## Abstract

**Background:**

Patients with end-stage renal disease (ESRD) are at a higher risk for chronic hepatitis, liver cirrhosis (LC) and mortality than the general population. Optimal modalities of renal replacement therapy for ESRD patients with concomitant end-stage liver disease remain controversial. We investigated the long-term outcome for chronic liver disease among dialysis patients in an endemic area.

**Methods:**

Using Taiwan’s National Health Insurance claim data (NHRI-NHIRD-99182), We performed a longitudinal cohort study to investigate the impact of comorbidities on mortality in dialysis patients. We followed up 11293 incident hemodialysis (HD) and 761 peritoneal dialysis (PD) patients from the start of dialysis until the date of death or the end of database period (December 31, 2008). A Cox proportional hazards model was used to identify the risk factors for all-cause mortality.

**Results:**

Patients receiving PD tended to be younger and less likely to have comorbidities than those receiving HD. At the beginning of dialysis, a high prevalence rate (6.16 %) of LC was found. Other than well-known risk factors, LC (hazard ratio [HR] 1.473, 95 % CI: 1.329-1.634) and dementia (HR 1.376, 95 % CI: 1.083-1.750) were also independent predictors of mortality. Hypertension and mortality were inversely associated. Dialysis modality and three individual comorbidities (diabetes mellitus, chronic lung disease, and dementia) interacted significantly on mortality risk.

**Conclusions:**

LC is an important predictor of mortality; however, the effect on mortality was not different between HD and PD patients.

## Background

The global prevalence and incidence of end-stage renal disease (ESRD) has been increasing annually [1,2]. The results of studies [3-8] on outcomes among dialysis patients appear to vary by country, follow-up period, age, baseline comorbidities, dialysis types and choice of study design.

The survival of hemodialysis (HD) and peritoneal dialysis (PD) seem inconclusive [8]. Age, type of dialysis [7], diabetes mellitus (DM), and other comorbidities need to be considered when estimating mortality among dialysis patients [3-5]. Elderly patients on PD are reported to have a poor prognosis [4-6], patients with diabetes receiving PD have been found to have a higher mortality than those receiving HD [3,4,6]. While Jaar BG et al. reported that ESRD patients who had higher propensity for initially receiving PD, survival did not differ by dialysis type [7]. Patients with ESRD are at a higher risk for chronic hepatitis, and thus are more likely to have higher rates of complications (LC and hepatocellular carcinoma) and higher mortality than the general population [9-16]. Taiwan is an endemic area for HCV. There, PD patients have a HCV prevalence rate of 10 % – 15 % [17,18], HD patients 15 % – 20 %, and the general population 5 % – 10 % [12,13]. Around ten percent of incident dialysis patients have been found to be positive for anti-HCV antibody [14], and 5.8 % of incident HD patients have been found to have LC [15] when beginning renal replacement therapy in Taiwan. Optimal modalities of renal replacement therapy for these patients also remain controversial [19-21]. Potential disadvantages of HD therapy are unstable hemodynamics and the risk of bleeding [19,22]. While PD therapy offers a more stable hemodynamic profile, it may increase the possibility of early catheter leak, peritonitis, and ongoing protein loss. HD has been reported to not prolong survival in LC patients with acute kidney injury (AKI), but not been carefully examined in those with maintained HD. While PD is also reported to be unhelpful in LC with AKI, but it has been found to produce viable results in some LC patients with ESRD [20].

We hypothesize that each comorbidity has a different impact on the long-term survival and mortality rates between HD and PD patients. The Asia-Pacific area is highly endemic for chronic liver disease [10,11,23]. However, published studies on this subject for Asian populations are scarce, especially epidemiological data for a national cohort of incident dialysis patients [6]. Using data from the Taiwan National Health Insurance (NHI) database form 1999 to 2008, we investigated the factors that may have an impact on mortality in dialysis patients.

## Methods

### Data sources

The National Health Insurance (NHI) program has provided compulsory universal health insurance in Taiwan since 1995. With the exception of prison inmates, all citizens are enrolled in the program. All medical institutions contracted with the NHI program must submit standard computerized claim documents for medical expenses. Patients with ESRD are eligible for any type of renal replacement therapy for free of charge; the expenses of chronic dialysis patients are covered by NHI.

Data for the study was obtained from the National Health Insurance Research Database (NHIRD) [Bureau of National Health Insurance. Available at: http://www.doh.gov.tw/statistic/index.htm [In Chinese] (accessed November 25, 2011); http://www.doh.gov.tw/EN2006/index_EN.aspx [In English], which was released for research purposed by the Taiwan National Health Research Institute (NHRI). This database covers nearly all (99 %) inpatient and outpatient medical benefit claims for Taiwan’s 23 million residents, making it one of the largest and most comprehensive databases in the world, and has been used extensively in various studies [24]. Patient identification numbers, gender, birthdays, dates of admission and discharge, medical institutions providing the services, the ICD-9-CM (International Classification of Diseases, 9th Revision, Clinical Modification) diagnostic (up to five) and procedure codes (up to five), and outcomes are encrypted. As the dataset was released with deidentified secondary data for public research purposes, the study was exempt from full review by the Institutional Review Board of Chi-Mei medical center and the Bureau of National Health Insurance (NHRI-NHIRD-99182). We used the NHIRD for all ambulatory care claims and inpatient claims from 1998 to 2008 for this study. All datasets can be interlinked through the individual’s unique personal identification number.

### Patient selection and definition

**Figure 1:**
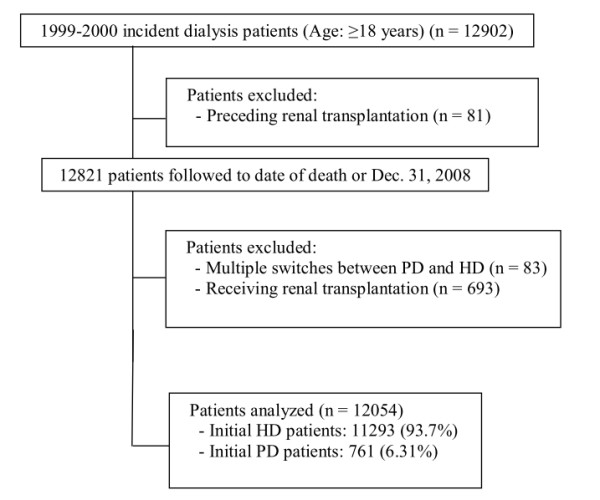
Flowchart showing incident dialysis population for evaluating long-term outcomes.

In this longitudinal cohort study, we selected all adult ESRD patients (≥18 years old) on maintenance dialysis who began renal replacement therapy between January 1^st^, 1999, and December 31^st^, 2000 (n = 12902). ESRD patients on maintenance dialysis were defined as receiving dialysis for more than 90 days [3,15], our indicator of a need for long-term dialysis. We excluded those who had undergone renal transplantation before beginning dialysis (n = 81). Patients were followed from the first reported date of dialysis to the date of death or December 31^st^, 2008, the end of the database period. We also excluded patients who received renal transplantation (n = 693) during the follow-up period or underwent multiple switches between HD and PD (n = 83). The determination of modality switches after the initial modality choice followed the “60-day rule” used by the USRDS (i.e., any change in modality lasting less than 60 days is not recorded as a “switch” in the database) [25]. In total, we analyzed data collected from 11293 incident HD and 761 incident PD patients (Figure 1).

### Ascertaining the demographic and comorbid variables

We linked to the diagnostic codes through the inpatient and outpatient claims databases of the NHI. Our data collection included not only patients` survival status, but also their date of death, demographics, and baseline comorbidities. Baseline comorbidities, including diabetes (DM), hypertension (HTN), congestive heart failure (CHF), coronary artery disease (CAD), cerebrovascular accident (CVA), peripheral arterial disease (PAD), chronic lung disease, liver cirrhosis (LC), cancer, dementia, and hemiplegia or paraplegia, are important factors affecting mortality and were assessed at the start of dialysis. These characteristics were consistent with those in previous studies [3,26] and demonstrate the need to adjust when comparing mortality rates among dialysis patients. The ICD-9-CM codes used to define each condition are shown in Table 1. Those comorbidities were identified according to one of the definitions below: (1) Diagnostic codes in outpatient visits if the patient had an initial diagnosis at any time the year leading up to beginning of dialysis and then experienced one or more additional diagnoses within the subsequent 12 months. The first and last outpatient visit within 1 year must had to be >30 days apart to avoid accidental inclusion of miscoded patients. [27,28] (2) Diagnostic codes in hospitalization databases at least one time within the year leading up to start of dialysis. The method of identifying these comorbidities have been used extensively in various studies of Taiwan National Health Research Institute and many articles have been published [Bureau of National Health Insurance. Available at: http://www.doh.gov.tw/statistic/index.htm [In Chinese] (accessed November 25, 2011); http://www.doh.gov.tw/EN2006/index_EN.aspx [In English].

**Table 1:** ICD −9-CM codes used to identify clinical conditions

**Conditions**	***ICD-9-CM***
Diabetes Mellitus	250.**, 357.2, 362.0*, 366.41
Hypertension	362.11, 401.*-405.*, 437.2
Congestive Heart Failure	398.91, 422, 425, 428, 402.*1, 404.*1, 404.*3
Coronary Artery Disease	410.**- 414.**
Cerebrovascular Accident/TIA	430-438.**
Peripheral Arterial Disease	440-440
Chronic Lung Disease	490-496*, 500-505*, 506.4*
Liver Cirrhosis	571.5, 571.6
Cancer	140-208; 230–231; 233-234
Dementia	290-290.9*
Hemiplegia or Paraplegia	344.1*, 342–342.9*

ICD-9-CM, *International Classification of Diseases, Ninth Revision, Clinical Modification*; TIA, Transient ischemic attack. * can be any number or missing.

### Statistical analyses

Parametric Pearson’s chi square test is utilized to compare each variable in HD and PD patients. Non-parametric tests were used for other analyses. The significance was set at *P* < 0.05.

Overall patient survival was described using the Kaplan-Meier method based on dialysis modality. Intent-to-treat analysis was analyzed using Cox proportional hazards model to identify the risk factors for all-cause mortality. Hazard ratios (HRs) and 95 % confidence intervals (CIs) were derived from Cox proportional hazards models. Cox models met the assumption of proportionality of risks. To adjust for potential confounding in the relationship between comorbidities and the risk of mortality, multivariate analyses were used to model to all-cause mortality. Further interactions were tested. The complete model, which included all the covariates, was used for cox regression analysis. Then, each interaction term was separately included once at a time. All statistical operations were performed using the Statistical Package for Social Sciences for Windows 17.0 (SPSS Inc; Chicago, IL, USA).

## Results

### Demographics and clinical characteristics

Initially, 11293 patients selected HD and 761 patients selected PD (Table 2). PD patients tended to be younger and to less likely have comorbidities than those receiving HD. Patients receiving PD were predominantly younger than those receiving HD (53.95 ± 15.09 years vs. 59.87 ± 13.45 years). During the follow-up period, 11216 patients (93.0 %) received pure HD treatment, 513 patients (4.3 %) received pure PD treatment, 77 patients (0.6 %) switched from HD to PD treatment, and 248 patients (2.1 %) switched from PD to HD. Many more HD patients than PD patients had DM and cardiovascular diseases, including CHF, CAD, and CVA. There were no significant differences between these two dialysis with regard to HTN, PAD, LC, cancer, dementia, and hemiplegia or paraplegia. Around six percent (6.2 %) of HD patients and 5.3 % of PD patients had LC.

**Table 2:** Patient characteristics and association with dialysis modality

	**PD (n = 761)**		**HD (n = 11293)**		***P*-value**
	n	(%)	n	(%)	
**Gender**					0.004
Female	445	(58.50)	5997	(53.10)	
Male	316	(41.50)	5296	(46.90)	
**Age (years)**					<0.001
18-44	217	(28.50)	1584	(14.00)	
45-64	347	(45.60)	5067	(44.90)	
≥65	197	(25.90)	4642	(41.10)	
**Cause of ESRD**					<0.001
Non-Diabetes Mellitus	478	(62.80)	5471	(48.40)	
Diabetes Mellitus	283	(37.20)	5822	(51.60)	
**Baseline Comorbidity**					
Hypertension					0.548
No	187	(24.60)	2667	(23.60)	
Yes	574	(75.40)	8626	(76.40)	
Congestive Heart Failure					<0.001
No	567	(86.30)	9172	(81.20)	
Yes	104	(13.70)	2121	(18.80)	
Coronary Artery Disease					0.01
No	614	(80.70)	8655	(76.60)	
Yes	147	(19.30)	2638	(23.40)	
Cerebrovascular Disease					0.002
No	698	(91.70)	9224	(87.90)	
Yes	63	(8.30)	1369	(12.10)	
Peripheral Arterial Disease					0.152
No	740	(97.20)	10867	(96.20)	
Yes	21	(2.80)	426	(3.80)	
Chronic Lung Disease					<0.001
No	716	(94.10)	10094	(89.40)	
Yes	45	(5.90)	1199	(10.60)	
Liver Cirrhosis					0.282
No	721	(94.70)	10590	(93.80)	
Yes	40	(5.30)	703	(6.20)	
Cancer					0.46
No	741	(97.40)	10831	(95.90)	
Yes	20	(2.60)	462	(4.10)	
Dementia					0.072
No	757	(99.50)	11150	(98.70)	
Yes	5	(0.50)	143	(1.30)	
Hemiplegia or Paraplegia					0.785
No	750	(98.60)	11143	(98.70)	
Yes	11	(1.40)	150	(1.30)	

PD: Peritoneal dialysis; HD: Hemodialysis; ESRD: End-stage renal disease.

### Cumulative survival rate

**Figure 2:**
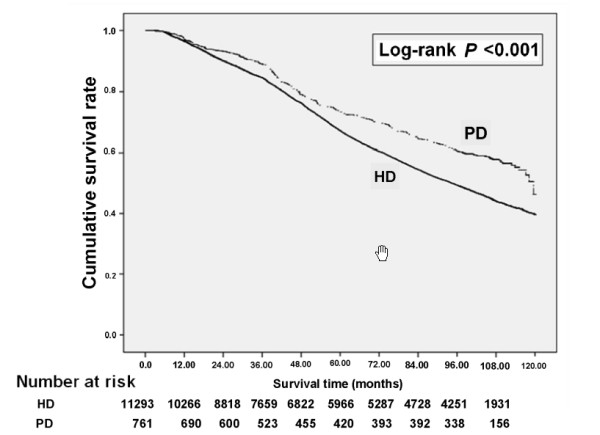
Crude overall survival curves for incident end-stage renal disease patients stratified by HD and PD at the start of dialysis.

During the follow-up period, 5374 patients died. The Kaplan-Meier survival curves for initial HD and initial PD patients can be viewed in Figure 2. For the PD group, mean follow-up time alive on dialysis was 91.39 months (95 % CI: 88.43-94.34), and for the HD group 83.20 months (95 % CI: 82.43-83.98). The cumulative survival rate of PD patients was 97.1 % at one year, 73.5 % at five years, and 57.8 % at nine years. The cumulative survival rate of HD patients was 96.5 % at one year, 67.2 % at five years, and 44.1 % at nine years. The differences in survival between PD and HD patients were significant (log-rank: P <0.001).

### Risk factors for all-cause mortality in all dialysis (HD and PD) patients

Based on a proportional hazards analysis performed to estimate the risk factors for all-cause mortality in dialysis patients, adjusted survival rates of HD versus PD patients were not significantly different (hazard ratio [HR] 0.882, 95 % CI: 0.776-1.001) (Table 3). There are several factors independently associated with mortality. Male patients had a higher mortality rate than female patients. Patients with DM, CHF, CAD, CVA, PAD, chronic lung disease, LC, cancer, dementia, and hemiplegia or paraplegia had a significantly higher mortality. However, patients with baseline HTN had lower mortality than those without HTN (HR 0.905, 95 % CI: 0.845-0.970). Further interactions between HTN and these comorbidities were tested. There was only one significant interaction: HTN × LC (P <0.001). After stratification, the effect of baseline HTN on lower mortality was much more prominent in LC patients (HR 0.726, 95 % CI: 0.573-0.920) than in non-LC patients (HR 0.924, 95 % CI: 0.860-0.933).

**Table 3:** Multivariable-adjusted model for all-cause mortality among dialysis patients

	**Univariate analysis**	**Multivariate analysis**
Covariate	HR (95% CI)	HR (95% CI)
Initial dialysis modality (HD *vs.* PD)	1.419 (1.251-1.609)*	0.882 (0.776-1.001)
Gender (Male *vs.* Female)	1.238 (1.174-1.306)*	1.192 (1.129-1.258)*
Age at initiation of Dialysis (years)		
18-44	Referent	Referent
45-64	2.710 (2.434-3.016)*	2.165 (1.942-2.415)*
≥65	5.709 (5.132-6.351)*	4.441 (3.979-4.956)*
Diabetes Mellitus (yes *vs.* no)	2.244 (2.124-2.371)*	1.841 (1.736-1.951)*
Hypertension (yes *vs.* no)	1.312 (1.228-1.401)*	0.905 (0.845-0.970)*
Congestive Heart Failure (yes *vs.* no)	1.969 (1.846-2.099)*	1.445 (1.348-1.549)*
Coronary Artery Disease (yes *vs.* no)	1.778 (1.675-1.888)*	1.127 (1.056-1.202)*
Cerebrovascular Disease (yes *vs.* no)	1.906 (1.766-2.057)*	1.303 (1.201-1.413)*
Peripheral Arterial Disease (yes *vs.* no)	1.829 (1.604-2.086)*	1.310 (1.147-1.496)*
Chronic Lung Disease (yes *vs.* no)	1.709 (1.575-1.855)*	1.222 (1.124-1.329)*
Liver Cirrhosis (yes *vs.* no)	1.612 (1.455-1.787)*	1.473 (1.329-1.634)*
Cancer (yes *vs.* no)	1.593 (1.405-1.807)*	1.368 (1.205-1.553)*
Dementia (yes *vs.* no)	1.910 (1.506-2.422)*	1.376 (1.083-1.750)*
Hemiplegia or Paraplegia (yes *vs.* no)	2.094 (1.700-2.580)*	1.445 (1.162-1.796)*

HR: Hazard ratio; CI: Confidence interval; *P <0.05.

Using all-cause mortality as the outcome, only three significant factors that interacted with dialysis mortality: DM, chronic lung disease, and dementia (Table 4).

**Table 4:** Interaction between dialysis modality and each comorbidity

**Interaction between dialysis modality and each comorbidity**	***P*-value**
Diabetes Mellitus × Dialysis modality	0.040
Chronic Lung Disease × Dialysis modality	0.025
Dementia × Dialysis modality	0.042

The complete model included all the covariates listed in Table 3 was used for cox regression analysis. Then, each interaction term was included separately once at a time.

### Risk factors for all-cause mortality among HD patients

We further stratified patients by dialysis modality. After multivariate analyses, we found an association between male gender and older age and higher mortality rates (Table 5). Baseline HTN was associated with lower mortality in HD patients (HR 0.904, 95 % CI: 0.843-0.971). All other baseline comorbidities were independent risk factors for higher mortality.

**Table 5:** Risk factors for All-Cause mortality among hemodialysis patients

	**Univariate analysis**	**Multivariate analysis**
Covariate	HR (95% CI)	HR (95% CI)
Gender (Male *vs.* Female)	1.224 (1.158-1.293)*	1.191 (1.127-1.258)*
Age at initiation of dialysis (years)		
18-44	Referent	Referent
45-64	2.682 (2.395-3.003)*	2.148 (1.915-2.409)*
≥65	5.583 (4.990-6.246)*	4.378 (3.903-4.910)*
Diabetes Mellitus (yes *vs.* no)	2.183 (2.063-2.310)*	1.821 (1.715-1.933)*
Hypertension (yes *vs.* no)	1.313 (1.228-1.405)*	0.904 (0.843-0.971)*
Congestive Heart Failure (yes *vs.* no)	1.919 (1.797-2.050)*	1.436 (1.338-1.541)*
Coronary Artery Disease (yes *vs.* no)	1.742 (1.639-1.853)*	1.125 (1.052-1.202)*
Cerebrovascular Disease (yes *vs.* no)	1.853 (1.715-2.003)*	1.298 (1.192-1.410)*
Peripheral Arterial Disease (yes *vs.* no)	1.815 (1.587-2.075)*	1.310 (1.144-1.499)*
Chronic Lung Disease (yes *vs.* no)	1.666 (1.533-1.810)*	1.209 (1.111-1.317)*
Liver Cirrhosis (yes *vs.* no)	1.612 (1.451-1.790)*	1.478 (1.330-1.643)*
Cancer (yes *vs.* no)	1.565 91.375-1.780)*	1.361 (1.195-1.549)*
Dementia (yes *vs.* no)	1.817 (1.425-2.316)*	1.431 (1.049-1.713)*
Hemiplegia or Paraplegia (yes *vs.* no)	2.085(1.682-2.584)*	1.462 (1.168-1.829)*

HR: Hazard ratio; CI: Confidence interval; *P <0.05.

### Risk factors for all-cause mortality among PD patients

Multivariate analyses revealed no significant differences in the risk factors for all-cause mortality between male and female PD patients (Table 6), though risk of mortality increased with age, especially in PD patients. Only old age, DM, CHF, and chronic lung disease were found to be independent risk factors for mortality in PD patients.

**Table 6:** Risk factors for All-Cause mortality among peritoneal dialysis patients

	**Univariate analysis**	**Multivariate analysis**
Covariate	HR (95% CI)	HR (95% CI)
Gender (Male *vs.* Female)	1.419 (1.110-1.814)*	1.202 (0.929-1.554)
Age at initiation of dialysis		
18-44	Referent	Referent
45-64	2.742 (1.939-3.876)*	2.113 (1.480-3.017)*
≥65	7.810 (5.363-11.373)*	5.224 (3.503-7.851)*
Diabetes Mellitus (yes *vs.* no)	3.198 (2.494-4.102)*	2.226 (1.700-2.914)*
Hypertension (yes *vs.* no)	1.216 (0.915-1.617)	0.944 (0.702-1.269)
Congestive Heart Failure (yes *vs.* no)	3.042 (2.201-4.204)*	1.746 (1.216-2.506)*
Coronary Artery Disease (yes *vs.* no)	2.413 (1.812-3.211)*	1.143 (0.825-1.583)
Cerebrovascular Disease (yes *vs.* no)	3.297 (2.239-4.856)*	1.481 (0.939-2.337)
Peripheral Arterial Disease (yes *vs.* no)	1.959 (1.007-3.814)*	1.320 (0.665-2.618)
Chronic Lung Disease (yes *vs.* no)	2.821 (1.782-4.467)*	1.819 (1.090-3.038)*
Liver Cirrhosis (yes *vs.* no)	1.527 (0.920-2.533)	1.424 (0.848-2.391)
Cancer (yes *vs.* no)	2.123 (1.159-3.889)*	1.290 (0.685-2.429)
Dementia (yes *vs.* no)	8.764 (2.764-27.790)*	3.473 (0.977-12.347)
Hemiplegia or Paraplegia (yes *vs.* no)	2.324 (0.958-5.638)	1.223 (0.462-3.240)

HR: Hazard ratio; CI: Confidence interval; *P <0.05.

## Discussion

This study used Taiwan NHI database, representing nationwide and representative population, to investigate long-term survival and mortality risk among dialysis patients. We found a high prevalence of LC among incident dialysis patients in our registry. The effects of some baseline comorbidities—DM, chronic lung disease, and dementia—on long-term mortality were not identical between HD and PD. Importantly, LC and dementia, in addition to the well-known risk factors, were predictors for mortality. In contrast, we found an inverse association between HTN and death.

Patients receiving PD had a better crude survival rate than those receiving HD. Those who selected PD were generally less likely to have comorbidities (Table 2). The HD group seemed to have a disproportionately higher number of the elderly patients. After adjustment, there was no statistical difference in terms of survival between the HD and PD groups. However, old age appeared to be the most important factor influencing survival in both groups. Patients aged ≥65 had a more than a 4-fold increase in mortality over those aged 18–44 (Table 3). We hypothesized that age might have confounded our finding of poor survival in the HD group. Using univariate analysis to estimate the risk factors for mortality, we found the two dialysis groups to have significantly different survival rates (HR 1.419, 95 % CI: 1.251-1.609) (Table 3). If we further adjust for mode of dialysis and age, the survival rate for HR went from 1.419 (95 % CI: 1.251-1.609) to 1.024 (95 % CI: 0.902-1.162), indicating that age confounded the finding of poor prognosis survival in the HD population. As seen in Figure 2, those receiving PD had varying rates of survival, while those receiving HD had almost linear pattern of survival. Several studies have reported that PD patients have better survival than HD patients during the first 2 years of dialysis [8,29,30]. The initial benefit of PD to patients may be found in fewer comorbidities, the removal of unidentified solutes by PD, or better preservation of residual renal function during this time period [29,30]. Having HD catheter may also be a reason for better initial survival of PD patients [31]. The concept of PD first and HD second implies that these two modalities of dialysis are complementary, and not in competition [8].

After the interaction test, three baseline comorbidities (DM, chronic lung disease, and dementia) interacted significantly with dialysis modality, indicating that these three factors had different impact on mortality between HD and PD patients. Similar to previous findings [3,4,11], the diabetes patients in our study had a higher mortality rate in the PD group (HR = 2.210) than those in the HD group (HR = 1.821). One study has reported that a diagnosis of dementia before the initiation of dialysis predicted subsequent death [32]. Likewise, we found our PD patients with dementia to be at greater disadvantage (HR = 3.473), the possible causes being peritonitis and their inability to do self-dialysis. Cavanaugh et al. [33] reported that patients who had chronic obstructive pulmonary disease were at greater risk of death in dialysis. Similarly, our multivariate analysis revealed that chronic lung disease to be an important predictor of mortality, even in patients on PD. We found a 21 % increase in the risk of death in patients on HD and an 82 % increase in those on PD. Therefore, it is important to individualize choice of dialysis modality. To do this, clinicians will need to consider the important factors that have different impact on survival between HD and PD -- DM, chronic lung disease, and dementia. In our study, ESRD patients with these three comorbidities at baseline had a higher mortality on those receiving PD than those on receiving HD.

After stratification, baseline comorbidities with cardiovascular diseases (CAD, CVA, and PAD), LC, and cancer were risk factors for death in HD patients, but not in PD patients. However, the HR estimates were remarkably similar, and interactions between treatment modality and these comorbidities were not significant. After the interaction test, there was a lack of significance because we believe PD group was too small to provide statistical power in our analysis.

Chronic liver disease and ESRD are serious common medical problems worldwide. Compared with the general population, ESRD patients are at increased risk of hepatitis B and C infection [10,11]. Period-prevalent data recorded in the national or regional dialysis registries of the ten Asia-Pacific countries/areas reveal the prevalence of HCV infection is considerably higher in dialysis patients than those in the corresponding general populations in many Asian countries (range 1.0–2.9 %) [23], and is likely to contribute to LC and death [16]. Around six percent (6.2 %) of our HD patients and 5.3 % of our PD patients had LC at the initiation of dialysis in Taiwan between 1999 and 2000, a prevalence which is much higher than that reported for western countries [30]. The treatment of ESRD patients with LC is complex and difficult, mainly due to deceased effective arterial volume and hemodynamic instability. The best dialysis modality for these patients is still controversial. Unstable hemodynamics and the risk of bleeding render HD problematic. Although PD has some disadvantages (early catheter leak, hypokalemia, peritonitis and ongoing protein loss), some reports still suggest that ESRD patients with LC can be successfully managed on PD [19,22], though we found no significant difference between HD and PD on all-cause mortality. De Vecchi et al. [34] reported dialysis patients with LC and those without liver disease had similar survival rates. In contrast, we found a 47 % higher risk of death in dialysis patients with LC than those without LC (HR 1.473, 95 % CI: 1.329-1.634).

The current study found that baseline HTN tended to be associated with decreased mortality among dialysis patients. The influence of blood pressure on the prognosis of dialysis patients is controversial [35-38]. While Salem et al. [37] suggested that antihypertensive treatment had a favorable effect on survival in dialysis patients, one recent study [36] reported an association between higher blood pressure and decreased mortality in dialysis patients without cardiovascular comorbidity. The results of our interaction test, supports the inverse association between HTN and mortality in non-DM patients (HR 0.810, 95 % CI: 0.739-0.889), but not in DM patients (HR 0.992, 95 % CI: 0.891-1.104). However, we cannot conclude that HTN per se or taking anti-hypertensive drugs produced the effect. Additional trials are required to investigate this issue. Some studies [38] found that arterial pressure can be a marker of organ failure. Our analysis showed that the effect of HTN on decreased mortality was much more prominent in LC patients than in non-LC patients. This may indicate that a higher arterial pressure predicts a lower rate of organ failure in LC patients on maintenance dialysis.

This study has several limitations. One limitation was that the comorbidity results relied on the claims data and ICD-9-CM diagnosis codes, which could potentially lead to disease misclassification. If these comorbidities were defined by ICD-9 codes only, then patients might have LC in several special situations, e.g., subclinically prior to dialysis, not coded for during outpatient ambulatory visit or hospitalization. Another limitation was that LC may or may not be related to viral hepatitis. However, we can’t have access to information about Hepatitis B or C positivity from this database. Still another limitation is one related to our use of billing data. This limited our access to of the body mass index, severity of comorbidities, and actual blood pressure values of the study population. Our study also lacked specific data on dialysis adequacy, type of vascular access used for HD patients, laboratory data, and medical prescriptions, which may affect survival particularly in patients with LC. Another might be our defining ESRD based on maintenance dialysis for more than 90 days. This lateness cause some early mortality related to LC to be missed. Finally, there may be some residual confounding as with all observational studies, and thus we can only show association, not causality, between these risk factors and mortality.

## Conclusions

In conclusion, this nationally representative study of incident dialysis patients found three baseline comorbidities, DM, chronic lung disease, and dementia, to have different effects on long-term outcomes in HD and PD patients. Taiwan is an endemic area for chronic hepatitis. This study showed that LC was an important predictor of mortality; however, the effect on mortality was not different between HD and PD patients. We found an inverse association between baseline HTN and subsequent mortality. In dialysis patients, a higher arterial pressure may indicate a lower rate of organ failure in patients with LC. Identifying high-risk patients at the start of dialysis might lead to more intensified treatment for these patients and thereby achieve better outcomes.

## Competing interests

The authors declare that they have no competing interests.

## Authors' contributions

CCC(Chien) collected data, analyzed, interpreted data and drafted the manuscript. JJW conceptualized the study and its objective, and also drafted the manuscript. YMS, SFW and CCC(Chu) extracted the data from the NIH databases, analyzed the data statistically. MJS collected data and provided clinical experience. DPS, CCC(Chio), HAC, JCH, YHL and HYW collected data, provided clinical experience, and revised the manuscript. WCK conceived the study, participated in the design, supervised the conduct of the study and helped to draft the manuscript. All authors read and approved the final manuscript.

## Pre-publication history

The pre-publication history for this paper can be accessed here:

http://www.biomedcentral.com/1471-2369/13/43/prepub
